# Dynamic behavior of a rotary nanomotor in argon environments

**DOI:** 10.1038/s41598-018-21694-2

**Published:** 2018-02-22

**Authors:** Kun Cai, Jiao Shi, Jingzhou Yu, Qing H. Qin

**Affiliations:** 1 College of Water Resources and Architectural Engineering, Northwest A&F University, Yangling, 712100 China; 2 Research School of Engineering, the Australian National University, Canberra, ACT 2601 Australia

## Abstract

When argon is used as a protecting gas in the fabrication or working environment of a nanodevice, absorption of some argon atoms onto the surface of the device lead to different responses. In this work, the rotation of the rotor in a carbon nanotube (CNT)-based rotary nanomotor in argon environment is investigated. In the rotary nanomotor, two outer CNTs act as the stator and are used to constrain the inner CNT (i.e., the rotor). The rotor is driven to rotate by the stator due to their collision during thermal vibration of their atoms. A stable rotational frequency (SRF) of the rotor occurs when the rotor reaches a dynamic equilibrium state. The value of the SRF decreases exponentially with an increase in the initial argon density. At dynamic equilibrium date, some of the argon atoms rotate synchronously with the rotor when they are absorbed onto either internal or external surface of the rotor. The interaction between the rest of the argon atoms and the rotor is stronger at higher densities of argon, resulting in lower values of the SRF. These principles provide insight for future experimentation and fabrication of such rotary nanomotor.

## Introduction

With the development of nanotechnology, the fabrication of nanodevices from lower-dimensional materials by mimicking macro-devices is becoming more and more realistic for accurate operation at nanoscale. The awarding in 2016 of the Nobel Prize in Chemistry to three scientists^1^ who had contributed much to the development of molecular machines in the latter part of last century seems a greater encouragement than the award itself. Besides chemical synthesis methods, physical approaches for the fabrication of nanodevices from lower-dimensional materials are worth investigating. In 2000, Cumings and Zettl^2^ found that inner carbon nanotubes (CNTs)^3,4^ withdrew back into fixed outer tubes when the inner tubes were released after being partially pulled out from the outer tubes. They also estimated the friction force and concluded that ultralow friction existed between neighboring tubes. On the basis of this property, they suggested making a linear nanobearing from CNTs. Inspired by that work, Zheng and Jiang^5^ proposed a physical model for a gigahertz oscillator made from CNTs. Their model was rapidly verified by molecular dynamics (MD) simulations^6,7^. In experiments in 2009, Somada *et al*.^8^ fabricated a linear bearing from CNTs, within which a CNT capsule could be driven to move back and forth in the host CNT.

Compared to linear translation, the operation of a rotary nanodevice faces greater challenges. For example, Fennimore *et al*.^9^ and Bourlon *et al*.^10^ separately fabricated a rotary nanobearing by using CNTs as the shaft to which a metal plate was attached, that was driven to rotate by an external electric field. Barreiro *et al*.^11^ observed both translation and rotation of a shorter CNT attached to a cargo on long CNTs that featured a thermal gradient along the tube axis. In the above models, the dimensions of the devices were not less than a hundred nanometers, far larger than those of a molecular machine in traditional concepts^12^. That is the major reason why the “nanomotor” faced criticism from chemists. Fortunately, a powerful tool, the MD approach^13,14^, met part of the requirements for the design of a nanomotor with sizes at the level of a few nanometers. Besides being adopted in the above works, the MD approach has also been widely used in the design of a rotary nanomotor. For instance, Kang and Hwang^15^ simulated the dynamic behavior of a carbon nanotube motor made from MWNTs when it was driven by a nanoliquid. Tu and Hu^16^ proposed a nanomotor model in which a short outer tube could move along the axis of a long inner tube, and the motion of the outer tube was driven by an external electric field along the tube axis. Using the electron tunneling property, Wang *et al*.^17^ designed a rotary nanomotor from carbon nanostructures, in which fullerene blades on a CNT-based shaft were charged/discharged periodically and driven to rotate. Although the size of the nanomotors involved in the above studies could be just a few nanometers, the auxiliaries were complex and difficult to miniaturize for fabrication.

**Figure 1:**
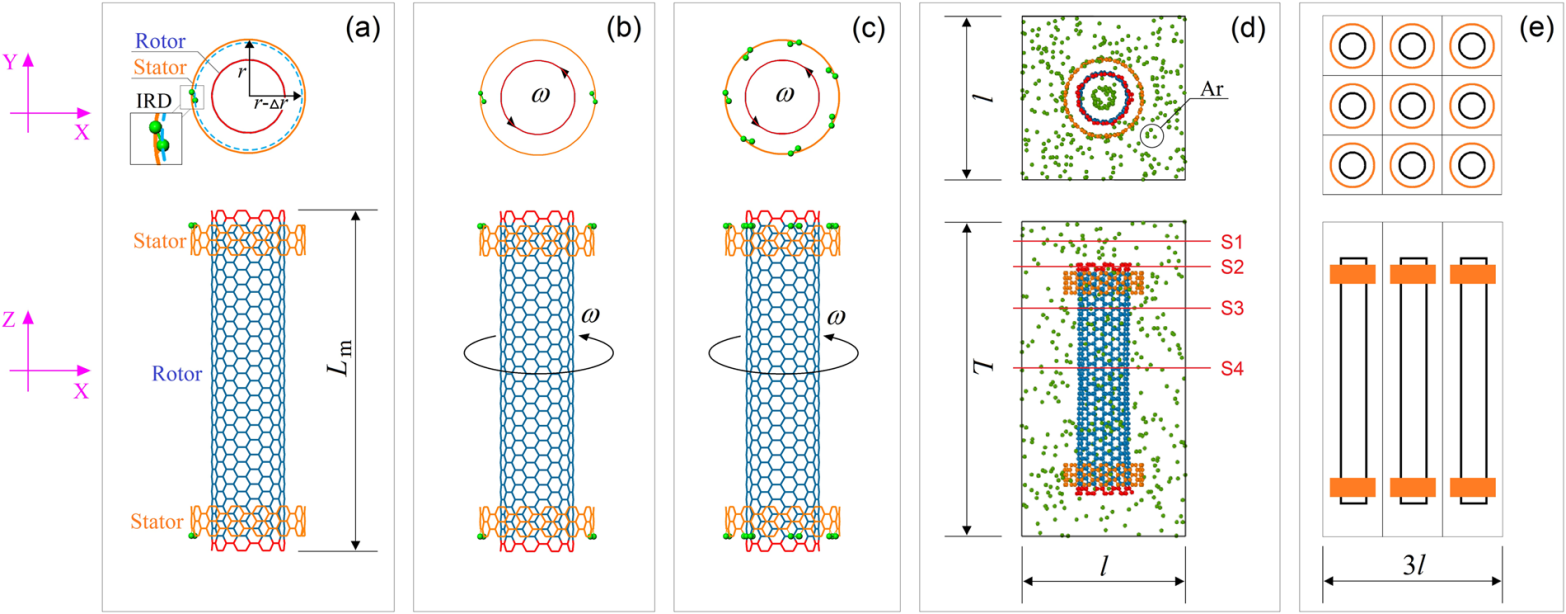
Schematic geometry of a thermally-driven rotary nanomotor made from (9,9)/(14,14) CNTs. (**a**) Each stator has IRD atoms (*N* = 1), (**b**) *N* = 2, and (**c**) *N* = 7. The rotational frequency of the rotor is labeled “*ω*”. In the X-Y plane, the radial deviation *∆r* = 0.4*l_C-C_ = ~0.0568 nm with *l*_C-C_ = ~0.142 nm. The initial distance along the tube axis between the neighboring ends of tubes is ~0.246 nm. The length of the inner tube (rotor) is *L*_m_ = ~5.657 nm. The length of each stator is ~0.492 nm. The radii of the (9,9) and (14,14) CNTs are ~0.610 and ~0.949 nm, respectively. (**d**) Layout of the simulation box with sizes *l* × *l* × *L*. The motor is placed at the center of the box that is filled with argon atoms. S1, S2, S3, and S4 are cross-sectional slices for observation of local atoms. (**e**) Schematic array of 3 × 3 nanomotors.

In 2014, Cai *et al*.^18^ reported their discovery of a new rotary nanomotor fabricated from CNTs. In their MD simulation, they found that the free inner tube could have a stable rotation at the frequency of ~100 GHz when the inner tube was put in an outer tube that was fixed after relaxation. The mechanism was briefly introduced: at a finite temperature the atoms on the rotor experienced dramatic thermal vibration. During the vibration, some atoms on the rotor might collide with fixed atoms on the stator. When the fixed atoms were laid out asymmetrically, they could provide tangential repulsion to the rotor during collision. That repulsion led to rotational acceleration of the rotor. Stable rotation of the rotor was approached when the friction between the rotor and the stator balanced the repulsion. In a thermally-driven rotary nanomotor (TRnM), the tubes could be less than 10 nm in length and the radii of the CNTs could be less than 1.5 nm. There was difficulty, however, in quantitatively controlling the rotation, until a new approach capable of solving this problem was presented^19^. In the work of ref.^19^, the stator was fixed symmetrically and one or more carbon atoms on the fixed edge of the stator were set to have an inwardly radial deviation (IRD) (e.g., Fig. 1a,b). As the collision between the rotor and the stator occurred, the IRD atoms provided stable repulsion to the rotor. Hence, the rotational direction of the rotor was determined when the IRD was specified. Recently, Cai *et al*.^20^ discussed the robustness of the rotational frequency of rotors after they overcame the difficulty of the rotational direction. Yang *et al*.^21^ estimated the significance of the major factors, namely temperature, IRD, and the diameter of rotor, on the output power of the TRnM.

In the above analyses, the TRnMs were situated in a vacuum. In general, nanomotors need to be fabricated or work in ambient environments. Since the discovery of CNTs, the interaction between many other types of atoms or molecules and CNTs has drawn much attention^22–27^. For example, CNTs can work as a nanochannel^28–32^ or even as a substrate to control the self-assembly of black phosphorus nanotubes from nanoribbon^33–35^. On the other hand, due to its chemical inertness, argon is usually used as a protective gas in experiments. Now, when a TRnM is placed in an argon environment^36,37^ rather than an absolute vacuum, the argon atoms may be attracted onto the CNTs and rotate together with the rotor. Due to the friction between the rotor and argon atoms and the improvement in the rotary inertia of the rotor, the dynamic response of the rotor may be obviously different from that of a rotor in a vacuum. In the present work, we discuss the dynamic behavior of a nanomotor array in argon environments, providing guidance for potential applications of rotary nanomotors in a nanomachine.

## Models and Methodology

### Models of nanomotor and simulation box

The geometry of a rotary nanomotor made from (9,9)/(14,14) CNTs in a simulation box is briefly introduced via Fig. 1. Typically, the definition of the IRD of an edge carbon atom on the outside of the stator is shown in Fig. 1a, namely, *∆r* for the deviation of the radius of the outer tube at the IRD atom. The two motors shown in Fig. 1b with *N* = 2 and in Fig. 1c with *N* = 7 are used in our numerical experiments. Figure 1d depicts a simulation box including a nanomotor and argon atoms. The boundary lengths of the box are *l* × *l* × *L*. Initially, we set the periodic boundaries of the simulation box to be −2 ≤ X ≤ 2 by −2 ≤ Y ≤ 2 by −1 ≤ Z ≤ 6.569, in nanometer units. Hence, *l* = 4 nm and *L* = 7.569 nm. In the box, the edge of the rotor is between [0, 5.657] nm. The volume of CNTs is ~16.827% of that of the box when the volume of CNTs is defined as *L*_m_ × (2*r*) × (2*r*). As we discuss the effects of box size on the rotational frequency of the rotor (*ω*), larger boxes will be involved in simulation. In each array, only 3 × 3 boxes are used (Fig. 1e).

To show the effects of the argon density in the box on the rotational frequency of the rotor, we set the density values from 100 kg/m^3^ to 1400 kg/m^3^ in the liquid state. The densities, together with the number of atoms and the corresponding pressures at 100 K, are listed in Table 1. The initial pressure is estimated using the ideal gas law^38^. In estimation, the effective volume of the gas is the difference between the volume of the box and that of the nanomotor.

**Table 1:** Initial states of argon in simulation box at 100 K. The initial pressure of a box with argon density of 100 kg/m^3^ at 100 K is ~30.5 bar. (In the present study, we set 1 bar = 1.013 × 10^5^ Pa).

Density/(kg/m^3^)	100	110	120	130	140	150	160	170	180	190	200	300
Num. of atoms	185	203	222	240	259	277	295	314	332	351	370	554
Density/(kg/m^3^)	400	500	600	700	800	900	1000	1100	1200	1300	1400	
Num. of atoms	739	923	1108	1293	1477	1662	1847	2031	2216	2401	2586	

### Flowchart of the MD simulation

To observe the dynamic behavior of the rotor in the argon-filled box, we adopt a MD simulation approach to perform the related numerical experiments. In each simulation there are four major steps:

1. Build the initial model (including CNTs and layout of argon atoms) according to the specified parameters. Initially, the argon atoms in the box are laid out regularly in lines;
2. Relax the system at a canonical (NVT) ensemble to obtain a reasonable layout of argon atoms. During relaxation, the carbon atoms on the stators are fully fixed, and the two ends (two rings at each end) of the rotor are fixed. The temperature is controlled using a Nosé-Hoover thermostat^14,39^;
3. After 1,500,000 steps of relaxation, release the fixed carbon atoms on the rotor and begin to record experimental data, namely, temperature, potential energy of system, rotational frequency of rotor, pressure, inertia moment of argon atoms about the tube axis.
4. Stop running after 10,000,000 steps of iteration.

In the simulation, the time step for integration of Newton’s second law of motion is 0.001 ps. The interactions among the atoms in the CNTs are evaluated by AIREBO potential^40^, and the interactions among argon/carbon atoms are estimated by the 12–6 type Lennard-Jones (L-J) potential^41^ with the relevant parameters of the L-J potential listed in Table 2. All simulations are carried out in the open source code LAMMPS^42^.

**Table 2:** Parameters in L-J potentials with respect to carbon and/or argon^49,50^.

Atom i	Atom j	*ε*_ij_(meV)	*σ*_ij_(Å)
C	C	2.4128	3.4000
Ar	Ar	10.3236	3.4050
C	Ar	4.9909	3.4025

To realize the system practically, four major steps are required: fix MWCNTs; tailor the CNTs into the model shown in Fig. 1; rearrange the IRD atoms at the external edges of stators, and finally, put the system in argon environment.

### Analysis of driving forces for the rotation of rotor

**Figure 2:**
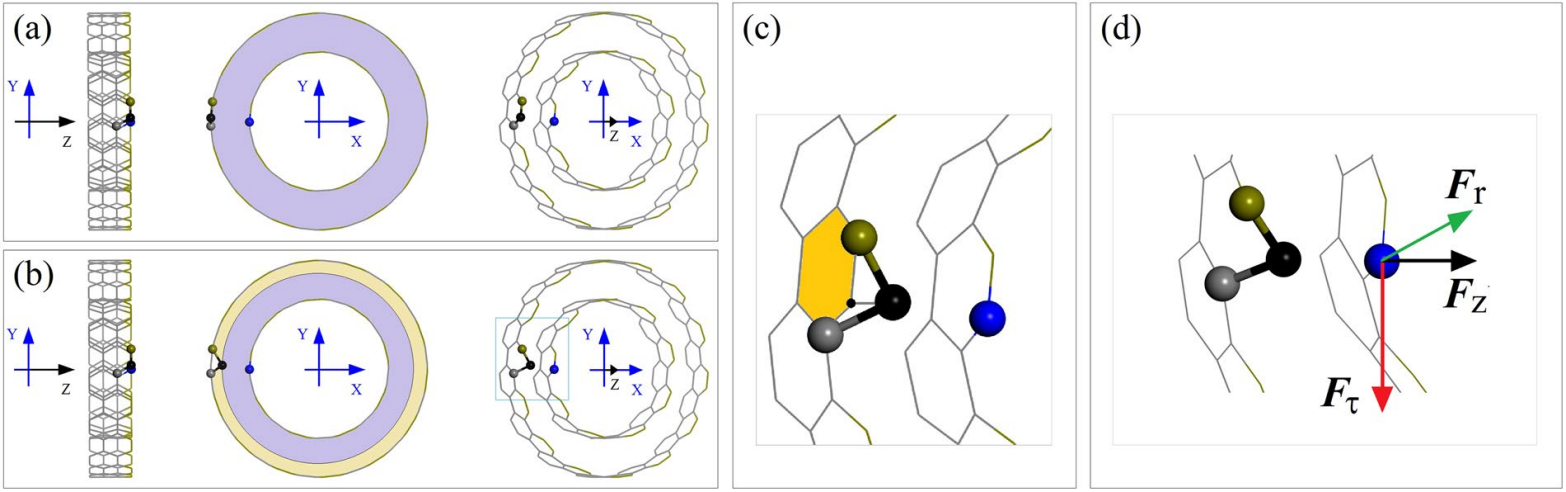
Schematic of IRD of an atom on the outer tube in double-wall CNTs (DWCNTs) with one unit along the axis. (**a**) Side, axial, and oblique views of DWCNTs without IRD of any atom. (**b**) The IRD is positive with the value equal to the width of the yellow zone. The black IRD atom moves closer to the inner tube, e.g., a blue atom on the inner tube. (**c**) Amplified local relative positions of atoms with or without IRD. (**d**) The components of repulsion on a blue atom subjected to the outer tube. *F*_τ_ and *F*_r_ are the tangential and radial components within the cross-section, and *F*_z_ is the component along the Z-/tube axis.

In Fig. 1a–c, the TRnMs with different numbers of IRD atoms are shown. The IRD of a carbon atom at the edge of a stator is marked with “∆*r*”, where *r* is the average radius of the stator. When the IRD is zero, the atoms at the edge of the stator are laid out symmetrically. If the IRD is non-zero, the symmetry disappears as all atoms on the stator are fixed. As we choose DWCNTs with the intertube distance of ~0.34 nm, i.e., equilibrium distance, a non-zero IRD means that the distance between the IRD atom and the rotor is less than the equilibrium distance (Fig. 2b). Hence, repulsion exists between the IRD atom and the rotor. The repulsion can be considered a pre-tightening force that will actuate the neighboring atoms on the rotor away from their original balance position. Observation of the relative positions of the covalent bonds between the IRD atom and its neighbor atoms shows that they are no longer located in-shell of the outer tube (Fig. 2c). Due to the layout of the bonds, the three components of the repulsion (Fig. 2d) are not simultaneously identical to zero. Among the three components, ***F***_τ_ determines the rotational acceleration of the inner tube. ***F***_r_ controls the breath-type vibration of the inner tube, and the axial relative sliding depends on ***F***_z_.

According to the foregoing analysis, the rotor’s rotation depends on the magnitude of ***F***_τ_. As the magnitude of time average is positive, i.e., anti-clockwise along the Z-axis, the component will generate a torque moment along the Z-axis that will drive the rotational acceleration of the rotor by obeying the moment of momentum theorem, i.e.,

1 $${M}_{z}={r}^{\ast }\times ({F}_{\tau }-{F}_{c})={J}_{z}\times \alpha ,$$

where *J*_z_ is the rotary inertia of the rotor with respect to the tube axis and *α* is the angular acceleration of the rotor along the Z-direction. r* is the average radius of both tubes at the IRD atom. *F*_c_ is the friction between two tubes. Hence, the rotational frequency of the rotor can be obtained by the integration:

2 $$\omega (t)=\frac{1}{2\pi }{\int }_{0}^{t}\alpha (s){\rm{d}}s.$$

By substituting Eq. (1) into Eq. (2), the rotational frequency of the rotor can be expressed as a function of ***F***_τ_ and r*:

3 $$\omega (t)=\frac{1}{2\pi }{\int }_{0}^{t}\frac{1}{{J}_{{\rm{Z}}}}[{r}^{\ast }\times ({F}_{{\rm{\tau }}}-{F}_{{\rm{c}}})]{\rm{d}}s.$$

Because the IRD is far less than the radius of the outer tube, r* differs only slightly from the mean value of the radii of both tubes. However, the repulsion on the rotor is very sensitive to the IRD according to the L-J potential. Hence, *F*_τ_ becomes obvious as IRD is in the interval [0.1, 0.4]*l*_C-C_. *F*_c_ also increases with the increase of IRD except for the friction between the rest of the atoms on the stator and rotor. When *F*_τ_ and *F*_c_ reach a balanced state, the rotational frequency of the rotor does not increase any further, and a stable rotational frequency (SRF) of the rotor is obtained.

When the system works at a finite temperature, the atoms on the rotor undergo dramatic thermal vibration and both the magnitude and the direction of their velocity depend on the temperature and conform to a Boltzmann distribution. Owing to the thermal vibration, the value of r* fluctuates noticeably and the repulsion between the IRD atom and the rotor varies simultaneously. At higher temperatures, the maximum value of repulsion increases and the friction between the two tubes also increases. Hence, at higher temperatures the SRF could be higher than at lower temperatures.

### Influence of argon atoms on the rotation of rotor

As the nanomotor is working in a zone filled with argon, two major factors need to be investigated for their influence on the interactions between the rotor and the argon atoms. One is the temperature of the system, which influences the interaction between the argon atoms and the rotor. For example, at low temperatures some atoms will be attracted by the inner tube and attach to surfaces of the rotor. Hence, the rotary inertia of the “new rotor” (CNT + Ar), i.e., *J*_z_, will be higher than that of the inner tube. That means that the rotational acceleration of the “new rotor” drops. If the system is at a higher temperature, the interaction could be reduced. The other factor is the argon density in the simulation box. As more argon atoms are introduced into the box, more argon atoms will be absorbed onto the surfaces of the CNTs at the same system temperature.

As some argon atoms are absorbed onto the surfaces of the rotor and stators, the rotation of the “new rotor” must be reduced by the rest of the argon atoms due to their relative motion that generates viscous resistance. Hence, the value of the SRF of the “new rotor” must be less than that of the CNT rotor in a vacuum. The influences of the density of argon and the interaction between argon atoms and CNTs are discussed with reference to the numerical results in the next section.

## Numerical Results and Discussion

### The SRF of a nanomotor in argon environments with different densities

**Figure 3:**
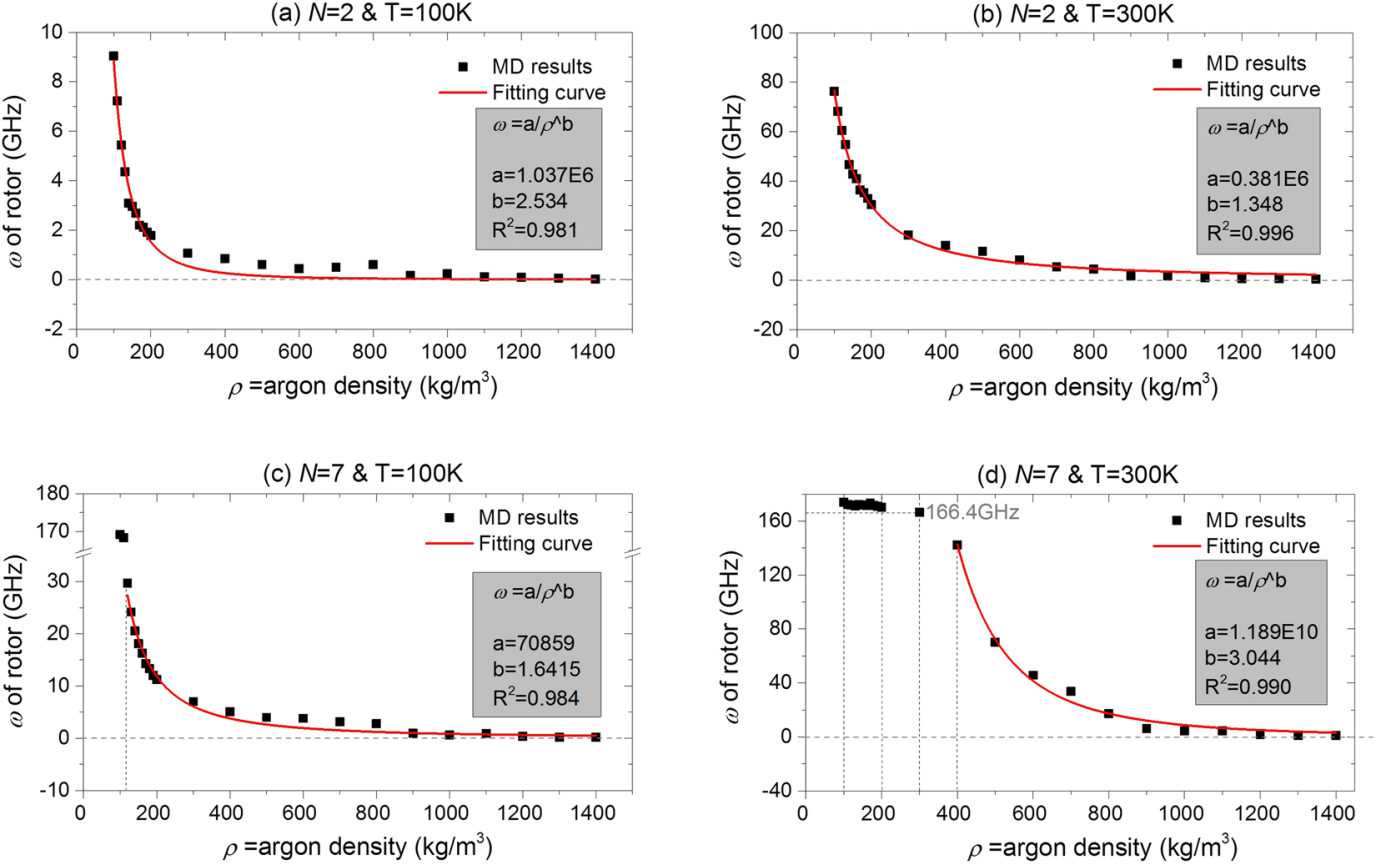
SRFs of rotor under different conditions. (**a**) A nanomotor with *N* = 2 is used, and the temperature (T) is 100 K, (**b**) *N* = 2 & T = 300 K, (**c**) *N* = 7 & T = 100 K, (**d**) *N* = 7 & T = 300 K.

To reveal the effect of temperature on the SRF of a rotor driven by different stators, four cases are considered, of stators with *N* = 2 and 7 at 100 K and 300 K, respectively. The temperature of 100 K is higher than the boiling point of argon at 1 bar, and 300 K is normal temperature. The relevant results are shown in Fig. 3 and Table 3. Without argon in the box, the SRF of the rotor is ~170 GHz. At any temperature, the value of the SRF of the rotor decreases with an increase in argon density. When *N* = 2 (with respect to cases a & b), the value of *ω* displays a quick drop when the argon density increases from 0 to 300 kg/m^3^. If the argon density increases further, the value of *ω* tends to be less than 1.0 GHz. For the same nanomotor and the same argon density, the value of *ω* of the rotor at 100 K is far less than that at 300 K. This result implies that it is possible to increase the SRF of the rotor by increasing the temperature and/or decreasing the density of the argon. As 1400 kg/m^3^ is the maximal density of the argon in a liquid state, the SRF of the rotor is still higher than 0.01 GHz (Movie 1). Hence, the nanomotor can work in an extreme environment, e.g., featuring low temperature and super-high pressure.

**Table 3:** SRFs of nanomotor in box filled with argon at different temperatures. (a) *N* = 2 & T = 100 K, (b) *N* = 2 & T = 300 K, (c) *N* = 7 & T = 100 K, and (d) *N* = 7 & T = 300 K. Units: *ρ* = Argon density: kg/m^3^, Rotational frequency: GHz.

*ρ*	0	100	110	120	130	140	150	160	170	180	190	200
(a)	169.0	9.038	7.229	5.434	4.368	3.097	2.965	2.689	2.201	2.112	1.920	1.778
(b)	*170.8*	76.24	68.28	60.49	54.87	46.66	42.88	41.02	36.38	35.32	33.02	30.51
(c)	*170.5*	169.2	168.4	**29.74**	24.17	20.53	18.14	16.32	14.27	13.33	12.06	11.21
(d)	*174.2*	*173.9*	*172.2*	*172.0*	*171.3*	*172.2*	*171.9*	*171.6*	*173.1*	*171.4*	170.8	*170.3*
***ρ***	**300**	**400**	**500**	**600**	**700**	**800**	**900**	**1000**	**1100**	**1200**	**1300**	**1400**
(a)	1.074	0.875	0.608	0.439	0.499	0.607	0.162	0.236	0.121	0.087	0.051	0.017
(b)	18.17	14.01	11.58	8.163	5.430	4.420	1.714	1.708	0.888	0.637	0.516	0.395
(c)	7.033	5.066	3.970	3.792	3.181	2.794	0.942	0.608	0.918	0.328	0.150	0.183
(d)	166.4	142.4	70.19	45.72	33.63	17.32	6.118	4.562	4.368	1.702	1.105	1.098

When *N* = 7 and T = 300 K, we find that the value of the SRF of the rotor is independent of the density of the argon (Fig. 3d) below 200 kg/m^3^. There are two reasons for this phenomenon. One is that it is difficult for the argon atoms at high temperature (300 K > 88 K of boiling point) with lower density to be attracted onto the CNTs. The other is that the stator has 7 IRD atoms that provide stronger power to drive the rotation of the rotor. However, if the density of the argon is greater than 300 kg/m^3^, the value of the SRF decreases rapidly.

**Figure 4:**
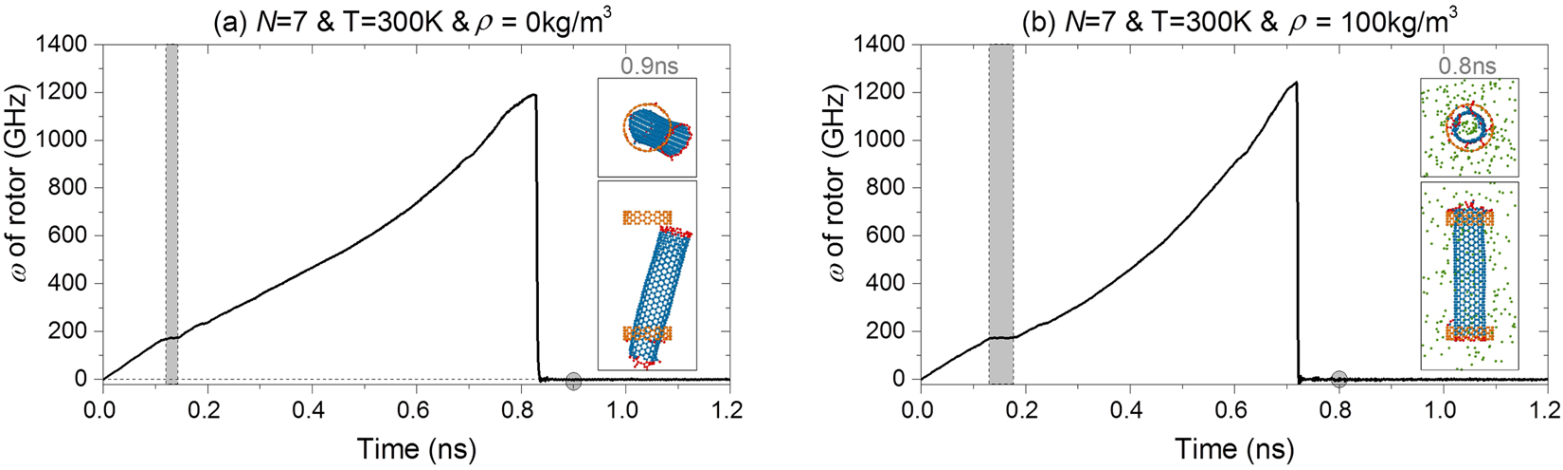
History of rotational frequency of a rotor in a nanomotor with *N* = 7 with or without argon atoms in the simulation box at 300 K. (**a**) Without argon atoms, (**b**) with argon density of 100 kg/m^3^.

It is necessary to demonstrate that the nanomotor may collapse due to covalent bonding between the rotor and a stator. Figure 4 gives the history of *ω* of a rotor driven by stators with *N* = 7 at 300 K. It is found that the rotational frequency of the rotor increases up to ~1200 GHz before sudden stoppage of the rotor in the box, with or without the presence of argon. But the differences are obvious. For example, when there is no argon in the environment, the rotor can escape from a stator and finally bond with a stator at its inner edge^43^. Meanwhile, the stoppage occurs near 0.83 ns if there is no argon. If the nanomotor is surrounded by argon atoms, both the final configuration of CNTs and the moment of stoppage of the rotor are different. From the results, we conclude that the nanomotor is not in a stable state when the rotational frequency of the rotor is very high. The rotor may display eccentric rotation at such high speed, that is the major reason for the escape of the rotor from its equilibrium state to finally bond with the stator^20^. In fact, the nanomotor collapses in many cases. In Table 3 the numbers in italics indicate that the nanomotor finally breaks down. The SRF of the nanomotor is obtained when it approaches the platform of the *ω* curve. As examples, the platform is indicated by a grey background in Fig. 4.

**Figure 5:**
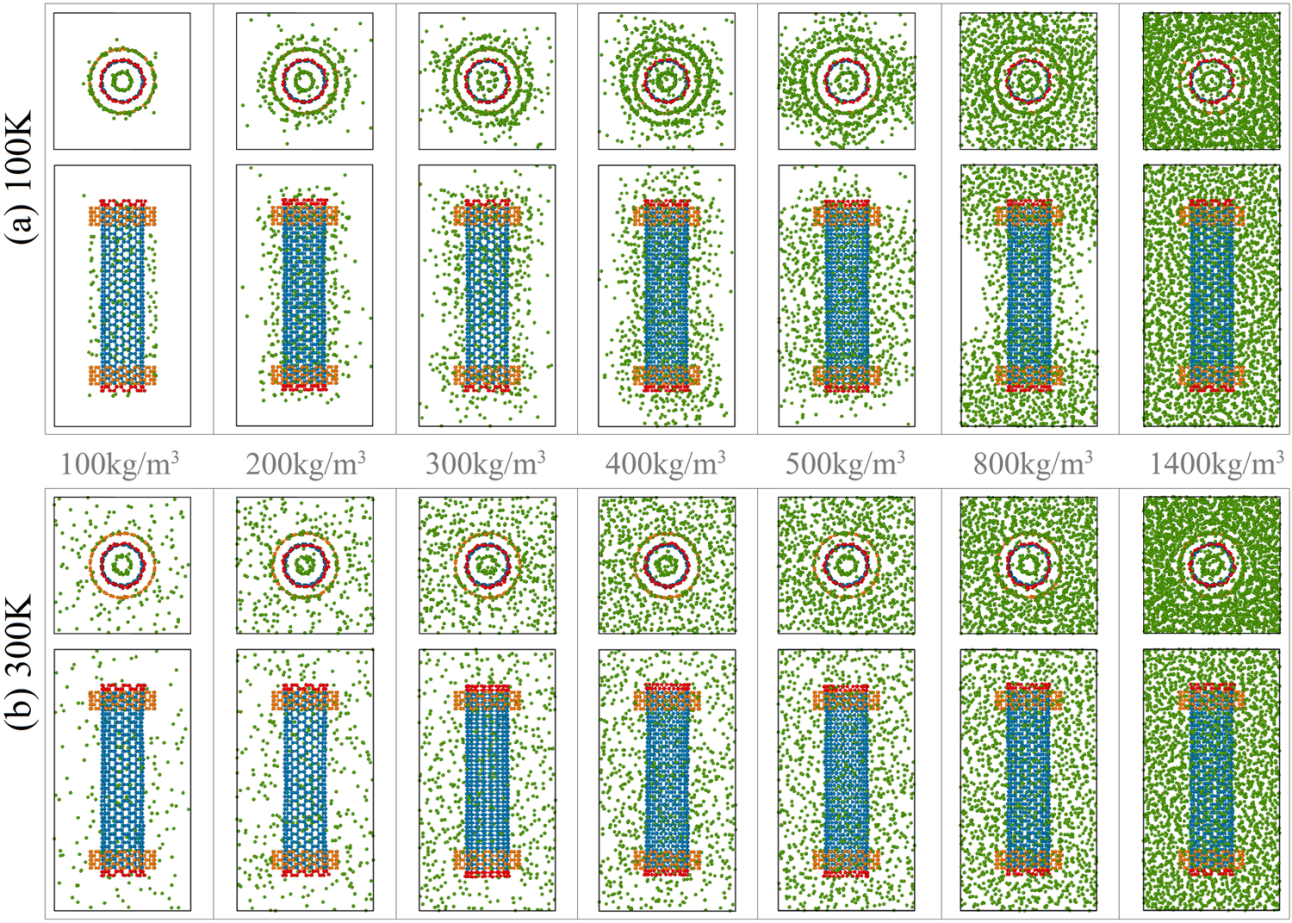
Representative configurations of the system in which the rotor is in a stable rotation state in argon environments at different temperatures. (**a**) At 100 K, the argon density is between [100, 1400] kg/m^3^, (**b**) at 300 K. In the system is the “*N* = 2” nanomotor.

From the above analysis, it is evident that, for the same nanomotor at different temperatures, the value of the SRF of the rotor is higher at higher temperature. As we capture representative snapshots of a system in which the rotor has stable rotation (Fig. 5a) at 100 K, we find that most of the argon atoms are attracted close to the CNTs at the distance of ~0.34 nm (Table 2). Some of the argon atoms even enter into or move out of the inner tube^36,37,44,45^. Due to the strong attraction, some of the argon atoms rotate together with the rotor. Hence, the rotary inertia of the “new rotor” becomes greater than that of the inner CNT^46–48^. Even when *ρ* = 400 kg/m^3^, the boundaries of the simulation box are very clear, i.e., few argon atoms are near the boundaries but more atoms are near the middle part of the rotor. These results mean that the pressure in the box in the stable state is far less than the initial pressure. As *ρ* = 500 kg/m^3^, the X-Z and Y-Z boundaries connect and the X-Y boundary is still clear. When *ρ* = 800 kg/m^3^, only the middle parts of the X-Z and Y-Z boundaries are clear. If the nanomotor is put into liquid argon, i.e., *ρ* = 1400 kg/m^3^, the distribution of the argon atoms appears uniform.

The situation changes when the system is at 300 K. For example, the argon atoms are distributed uniformly in the box and few argon atoms attach to the surfaces of the CNTs (Fig. 5b). This result means that the friction between the rotor and the argon atoms (Eq. (3)) is lower at 300 K than that at 100 K. This is the major reason for the obvious difference between the stable rotational frequencies of the rotor at different temperatures (Table 3). Considering this reason, we mainly discuss the dynamic behavior of the system at 100 K in the following sections.

**Figure 6:**
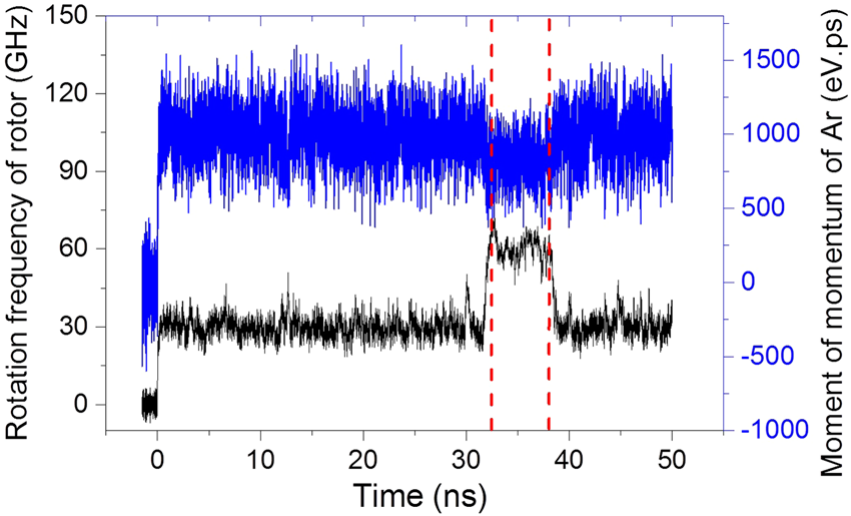
Histories of the rotational frequency of the rotor and the moment of momentum of argon in the system with the *N* = 7 nanomotor at 100 K. The initial argon density is 120 kg/m^3^.

When we observe the history curve of the rotational frequency of a rotor, e.g., *N* = 7, T = 100 K, and the initial argon density is 120 kg/m^3^, the frequency remains stable for about 31 ns before suddenly jumping up to ~60 GHz. The rotational frequency of the rotor stays the same for about 6 ns after the jump, but finally falls to the level observed before the jump, i.e., the value of *ω* is close to 29.74 GHz (black line in Fig. 6). As determined from the blue line in Fig. 6, the moment of momentum of argon atoms about the tube axis undergoes an obvious decrease during the same period. Hence, we conclude that the argon atoms undergo sliding relative to the outer surface of the rotor. Before the relative sliding occurs, the argon atoms attached to the outer surface of the rotor rotate synchronously with the rotor. But during the relative sliding, the group of argon atoms rotates more slowly than the rotor (Movie 2). After about 6 ns of relative sliding, they rotate synchronously, again. This phenomenon is evidence of friction between the rotor and the argon atoms.

**Figure 7:**
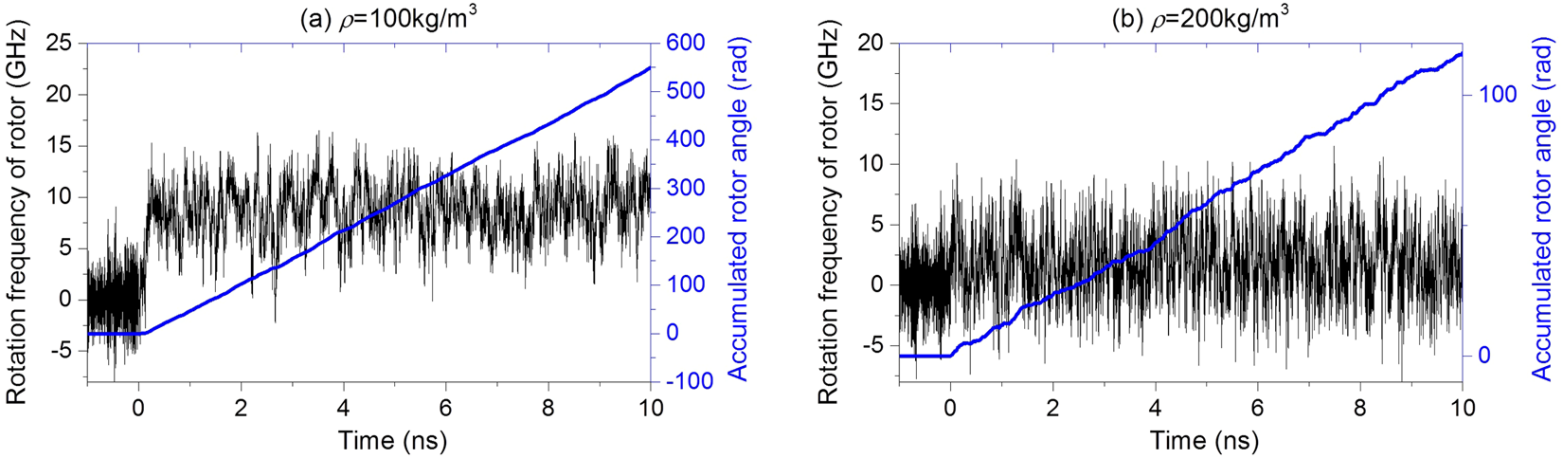
Histories of the rotational frequency and the accumulated angle of rotor in the nanomotor with *N* = 2 at 100 K in argon with different densities. (**a**) Argon density is 100 kg/m^3^. (**b**) Argon density is 200 kg/m^3^.

In Table 3, most of the values of *ω* are below 10 GHz, or even below 1.0 GHz. The fluctuation of the rotational frequency of the rotor is usually greater than 10 GHz. Hence, to obtain the SRF of the rotor, we first calculate the accumulated rotary angle of the inner tube under such conditions in simulation. The value in Table 3 is obtained by the averaged time of the accumulated angle. For example, the rotational history of the rotor driven by the *N* = 2 stators at 100 K is given in Fig. 7. Compared to the large fluctuation of rotational frequency, the accumulated rotor angle changes only slightly and is in almost a straight line. The values of the SRFs listed in Table 3 are equal to the slope of the straight line. In the following analysis, the history of the accumulated rotor angles is used.

### Effect of size of simulation box on SRF of nanomotor

In previous discussion, the size difference between the nanomotor and the simulation box is low, a feature that influences the statistics of the pressure at the boundary. For example, the radius of the outer tube (stator) is ~0.949 nm, indicating that the distance between the tube and the X-Z or Y-Z boundary of the box is only ~1 nm, i.e., the cutoff of the L-J potential. At 100 K, due to the strong attraction of the tube to the argon atoms, most of the argon atoms attach to the surface of the rotor (e.g., Fig. 5a). The pressure in the box, estimated by substituting the volume of the box that does not include the space occupied by the nanomotor plus the number of argon atoms in it into the ideal gas law equation, shows large fluctuation during simulation. Hence, we do not calculate the pressure. To obtain relatively reasonable statistics of pressure, we expand the original simulation box by setting the periodic boundaries to be double or 3 times their original values. Thus the volume of the simulation box becomes 2^3^ = 8 or 3^3^ = 27 of the initial volume, respectively.

To show the effect of the friction between the argon atoms and the rotor, we set the nanomotor with *N* = 2 at 100 K. The nanomotor is always placed symmetrically at the center of the box. To indicate the influence of the argon density on the dynamic response of the rotor, we choose 6 different values for the argon density, i.e., 50, 100, 200, 300, 400, and 600 kg/m^3^.

#### Each side of simulation box is double its original value

**Figure 8:**
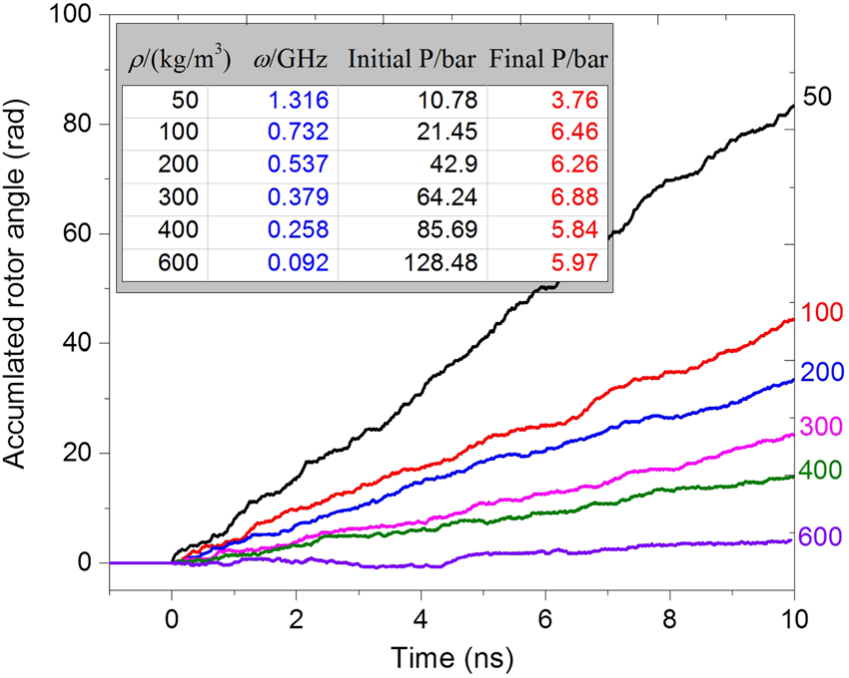
Histories of the accumulated rotor angles in the box with *l* = 8 and with different argon densities. The other three quantities, i.e., time averaged rotational frequency of the rotor, initial and final pressure (P) of the box, are given in the inserted table.

In this case, the periodic boundaries of the new box are set as −4 ≤ X ≤ 4 by −4 ≤ Y ≤ 4 by −4.828 ≤ Z ≤ 10.485, with the unit of nanometer. Hence, the width of box *l* = 8. The volume of CNTs is ~2.103% of that of the box. To keep the same initial argon density in the box, the number of argon atoms is 8 times that in Table 1. For example, there are 8 × 185 = 1480 argon atoms in the box with the initial argon density of 100 kg/m^3^. Figure 8 gives the history of the accumulated angle of the rotor within 10 ns. From the inserted table in Fig. 8, the rotational frequency of the rotor decreases when the argon density increases. The final pressure of the simulation box is nearly 6 bar when the density is greater than 50 kg/m^3^ at which the final pressure is ~3.76 bar. Obviously, the pressure in box is still much higher than the standard atmospheric pressure, for two reasons. One reason is that the temperature of the system is 100 K, higher than the boiling point of argon at the standard atmospheric pressure. The other reason is that not all of the argon atoms are attracted close to the CNTs and the rest of the argon atoms generate the high pressure.

**Figure 9:**
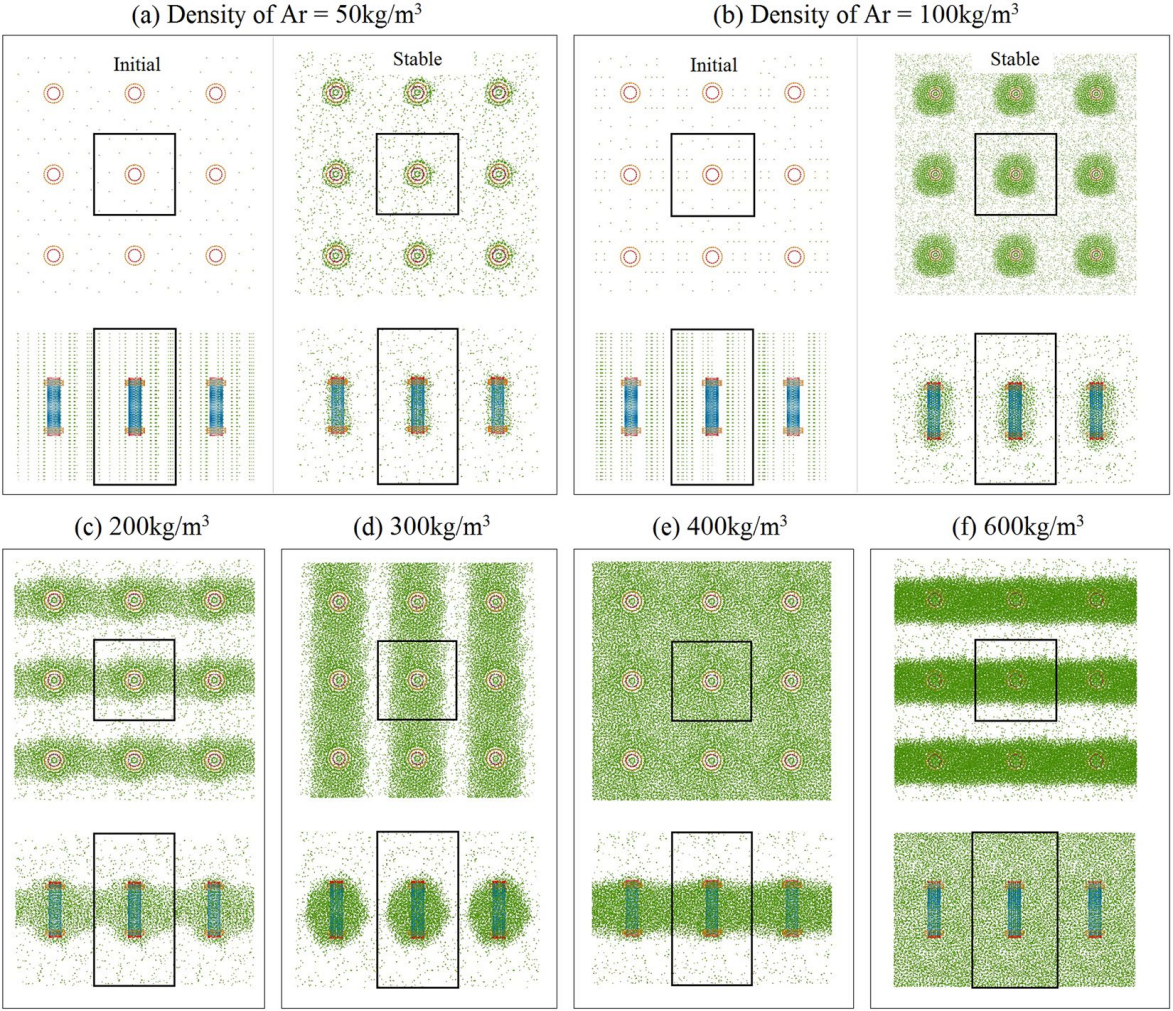
The configurations of the stable system with different densities of argon in the 3 × 3 boxes at 100 K. The inserted rectangles are the boundaries of a simulation box. In (**a**) and (**b**), both the initial and the stable states of the system are given. In (**c**–**f**), only the stable state is shown.

From Fig. 9 it can be seen that the final distributions of argon atoms in the simulation box differ due to differences in density. For example, only some argon atoms are attracted close to the CNTs with 0.5 nm when the initial argon density is 50 kg/m^3^ (Fig. 9a). When the initial argon density reaches 100 kg/m^3^, the rotor is covered by argon atoms to the thickness of over 1 nm (Fig. 9a). In that case, all the boundaries of the box have only a few argon atoms attached, whose motions contribute to the pressure of the box. When we increase the argon density in the box, argon clusters appear. For example, clusters are connected between the neighboring periodic boxes along the X-direction when the argon density is 200 or 600 kg/m^3^. The only difference is that the thickness of the cluster along the Z-direction in Fig. 9f (with respect to 600 kg/m^3^) is equal to the length of box along the same direction. The layout of the argon clusters in the 3 × 3 boxes resembles a sandwich structure along the Y-direction. However, the clusters in the 3 × 3 boxes with the initial density of 200 kg/m^3^ appear like heavy wires along the X-direction. When the initial density is 300 kg/m^3^, the argon clusters in the 3 × 3 boxes (Fig. 9d) resemble heavy wires along the Y-direction. Because the initial simulation box including all atoms is symmetric about the X-Y plane and the axis of the stators is aligned with that of the box along the Z-direction, the final layout of the argon clusters is random along either the X- or Y-direction.

When the initial argon density is 400 kg/m^3^ (Fig. 9e) (Movie 3) there is only one argon cluster, which is similar to a plane parallel to the X-Y plane. Hence, the presence of the CNTs leads to condensation of the argon atoms. As we place the rotary nanomotor in argon gas with the initial pressure of 1 bar at 100 K, we predict that the final layout of the argon atoms near the nanomotor will be approximately identical to that in Fig. 9a. This outcome means that the SRF of the rotor will be far less than that of the rotor in a vacuum, e.g., ~1.316 GHz in this case. On the other hand, because we choose argon as the protective gas in the operation of the nanomotor, the rotational frequency of the rotor will depend heavily on the value of *N*, as shown in Table 1, i.e., stators with lower numbers of IRD atoms will generate a lower rotational frequency of the rotor.

#### Each side of the simulation box is 3 times its original value

Now, we choose a larger simulation box for the nanomotor and argon atoms, setting the boundaries as −6 ≤ X ≤ 6 by −6 ≤ Y ≤ 6 by −8.657 ≤ Z ≤ 14.314, with the unit of nanometer. Hence, *l* = 12 and the volume of CNTs is ~0.623% of that of the box. Hence, the size effect can be ignored because a box larger than the present one is chosen in simulation. The initial argon density in the box is set at 50 or 100 kg/m^3^ to show the influence of box size on the rotor’s SRF.

**Figure 10:**
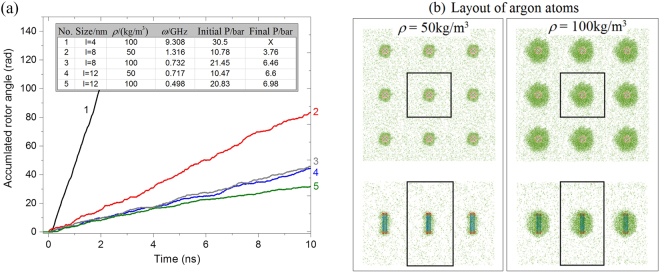
Comparison of MD results. (**a**) Histories of the accumulated angle of rotor in different boxes with different argon density. The related rotational frequency of the rotor and pressure (P) of the box are in the inserted table. The “size” in the inserted table is the width of the box. The nanomotor with *N* = 2 is used. (**b**) Layout of the argon atoms in the box with *l* = 12 and the rotor in stable rotation.

To demonstrate the size effect, the histories of the accumulated angles of the rotor in the same nanomotor (*N* = 2) in boxes with initially low argon density are shown in Fig. 10, in which the inserted table provides the related results of the system. As an example, when the initial densities of argon in the boxes are the same, i.e., 100 kg/m^3^, the SRFs of the rotor are obviously different. For example, in the original small box with *l* = 4, the final SRF of the rotor is ~9.3 GHz. However, when the box is larger, e.g., with *l* = 8, the value of *ω* is ~0.73 GHz, slightly higher than the values of the rotor in the largest box with *l* = 12. When the argon density in the boxes is set at 50 kg/m^3^, the value of *ω* of the rotor in the box with *l* = 8 is ~1.32 GHz, higher than that of the rotor in the box with *l* = 12. As evident from the differences among the final pressures of the boxes, the rotor attracts more argon atoms in the stable state of boxes with higher argon density. For example, in the same box with *l* = 12, the value of *ω* is ~0.72 GHz at *ρ* = 50 kg/m^3^, whereas *ω* is ~0.5 GHz at *ρ* = 100 kg/m^3^. The prediction is verified by the layout of the argon atoms in the final stable state of the box as shown in Fig. 10b. On the other hand, due to the similar final pressure in the box, the number of argon atoms attracted close to the rotor in the box with *ρ* = 100 kg/m^3^ is far greater than that of the rotor in the box with *ρ* = 50 kg/m^3^. However, the difference of *ω* with respect to different argon densities is not obvious. Hence, from the moment of momentum theorem we conclude that the number of argon atoms that rotate synchronously with the rotor depends slightly on the argon density. The rest of the argon atoms clearly display relative sliding with the “new rotor”, resulting in a lower value of *ω*.

### Effect of length of rotor on the value of SRF

In previous discussion, nanomotors of the same size were used. In fact, the size of the CNTs has a significant influence on the SRF of the rotor. For convenience of comparison, we again choose CNTs in the nanomotor with sizes between (9,9) and (14,14). The stators have the same configurations, i.e., the same length and *N* = 7. But the length of the rotor is different from the original value, ~5.657 nm. Currently, we choose 0.5 × *L*_m_, 1.0 × *L*_m_, 1.5 × *L*_m_ and 2.0 × *L*_m_ as the rotor length in simulation. Due to the use of a longer rotor, we choose a larger box, whose periodic boundaries are set as −4 ≤ X ≤ 4 by −4 ≤ Y ≤ 4 by −4.828 ≤ Z ≤ 10.485, with the unit of nanometer. Five densities of argon are involved in the simulation, namely 200, 300, 400, 500, and 600 kg/m^3^.

**Figure 11:**
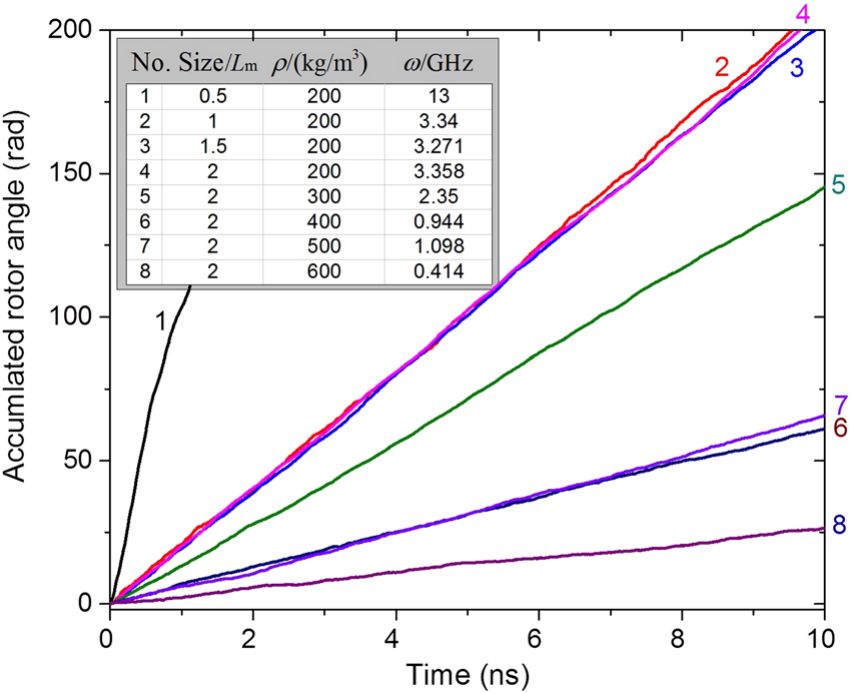
Histories of the accumulated rotor angles in the same boxes (*l* = 8) with different argon density.

**Figure 12:**
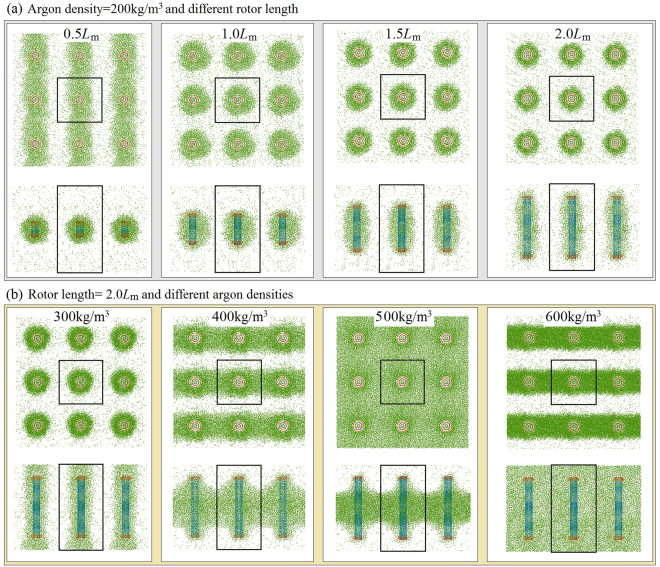
Stable states of simulation boxes in which nanomotors are surrounded by argon atoms. (**a**) In boxes with the same argon density, the rotor lengths differ. (**b**) For the same rotor in the boxes with different argon densities.

Figure 11 gives the history curves of the accumulated rotor angles in eight cases, as listed in the inserted table. When rotors with different lengths are placed in boxes with the same argon density, e.g., 200 kg/m^3^, their SRFs differ. For example, for the shortest rotor, with length 0.5 × *L*_m_, the SRF is about four times of that of the rotor with length of 1.0 × *L*_m_. The reason is that fewer argon atoms are attached to the shorter tubes and the interaction among the CNTs and argon atoms becomes weaker, as shown in Fig. 12a. Driven by the same stators, the shortest rotor is subject to the lowest friction from the argon atoms.

However, when a longer rotor is in the gas, more argon atoms attach to the surface of the rotor, and furthermore, both the rotary inertia of the “new rotor” and the friction between the rest of the argon atoms and the “new rotor” increase quickly and control the maximum value of the SRF. From both the curves labeled “2, 3, and 4” and the statistical values of *ω* in the inserted table, the SRFs differ slightly. The differences are caused by two main factors. One is that the boxes contain the same number of argon atoms, and most of the gas atoms are attracted onto the surfaces of the CNTs. Due to lower number of argon atoms, the boundaries of the box in the stable state are clear, i.e., only a few argon atoms can be found near the boundaries. This result implies that neighboring rotors in different boxes have no interaction via the gas cluster. Hence, the local state of a rotor depends only on the final layout of argon atoms in its box. If, for example, when the argon density is higher, i.e., 300 kg/m^3^, an interaction exists with a neighboring rotor along the Z-direction, that increases the friction between argon atoms and rotor. The second factor is present when a rotor of any length is fully covered by argon atoms, and the interactions between the rotor and argon atoms are similar according to the layout of argon atoms within 0.5 nm of the outer surfaces of the CNTs, especially at the neighboring ends of CNTs. It is known that the final SRF of a rotor approaches only when the rotor is in an equilibrium state, which contains the rotational equilibrium along the Z-direction. When most of the argon atoms are within 1.0 nm of the neighboring surfaces of the rotor, most of the argon atoms can be driven to move with the rotor while the rest of the argon atoms prevent the motion, i.e., they provide friction on the “new rotor”. Meanwhile, the rotor is driven by the same stators, i.e., the active force on the rotor is identical for the three cases. Hence, the equilibrium state of the “new rotor” depends on the relative sliding between the “new rotor” and the rest of the atoms. Because the rotors of different lengths have the same environment in a stable state, their SRFs differ slightly.

Because we placed the longest rotors with length 2.0 × *L*_m_ in boxes with different argon densities, the SRF of the rotor in lower argon density is generally higher. The phenomenon is similar to that shown in Figs 8 and 9. The SRF of the rotor in the argon with the density of 500 kg/m^3^ is slightly higher than that in the argon with the density of 400 kg/m^3^. The major reason is that fewer argon atoms are attracted onto the rotor adjacent to the edges of stators when *ρ* = 500 kg/m^3^. Hence, the friction between the new rotor and the other argon atoms is lower, leading to a higher SRF.

## Conclusions

The dynamic response of the rotor in a nanomotor in an argon environment is investigated using MD simulations. Some typical factors, namely temperature, initial argon density, size of box, and length of rotor, are considered in experiments. From the numerical results, some concluding remarks are drawn as follows:

1. The SRF of a rotor decreases exponentially with the argon density. At lower temperatures, it decreases more quickly. At 100 K, the rotor can still rotate in liquid argon. At normal temperature, the SRF value of depends slightly on the argon density being less than 200 kg/m^3^;
2. At 100 K, in the box with lower argon density, most of argon atoms are absorbed near to the inner and outer surfaces of the rotor and rotate with the rotor. That is one of the reasons for the decrease in the rotational frequency of the rotor;
3. At normal temperature, the argon atoms distribute uniformly in the simulation box, and the friction between the rotor and the argon atoms is lower than that at 100 K because fewer argon atoms are absorbed onto the CNT surfaces;
4. The argon atoms on both surfaces of the rotor may slide relative to the rotor even after a long period of stable synchronous rotation;
5. For the same nanomotor in boxes with the same argon density but different dimensions, the SRF of the rotor in a larger box is lower, due to the greater number of argon atoms attracted onto the CNTs.
6. In the same box the same low argon density, the SRF of a rotor depends slightly on the tube length when the length is greater than 5 nm.

According to the interaction between the nano-flow of argon and the CNTs, we know that the argon atoms can also transit kinetic energy onto the CNTs. One can heat the rotors to rotate by introducing heated argon. Therefore, the present model is different from that in Cai *et al*.^18,19^.

## Electronic supplementary material

Supplementary information

Movie 1

movie 3

movie 2

Explanations for Movies
