# Supplementary material for: Dynamic behavior of a rotary nanomotor in argon environments
Source: Sci Rep. 2018 Feb 22;8:3511. doi: 10.1038/s41598-018-21694-2 (PMC5823920; doi:10.1038/s41598-018-21694-2)
Supplement: Supplementary file 1 — Supplementary information [file 41598_2018_21694_MOESM1_ESM.pdf]

Dynamic behavior of a rotary nanomotor in argon environments

Kun Cai [1, 2*], Jiao Shi [1], Jingzhou Yu [1], Qing H. Qin [2*]

[1] *College of Water Resources and Architectural Engineering, Northwest A&F University, Yangling 712100, China*
[2] *Research School of Engineering, the Australian National University, ACT 2601, Australia*

*Corresponding authors' email address: Kuncai99@163.com (Kun Cai); qinghua.qin@anu.edu.au (Qinghua Qin)

Supporting materials

1. Movies
   - Movie 1--roi=1400-N=2-T=100K during [10, 14]ns
   - Movie 2--roi=120-N=7-T=100K during [26, 26.1]ns at left, [31.9, 32]ns at right
   - Movie 3--roi=400-N=2-T=100K during [10, 11]ns

2. Explanations for Movies.doc
