# Supplementary material for: Dynamic behavior of a rotary nanomotor in argon environments
Source: Sci Rep. 2018 Feb 22;8:3511. doi: 10.1038/s41598-018-21694-2 (PMC5823920; doi:10.1038/s41598-018-21694-2)
Supplement: Supplementary file 5 — Explanations for Movies [file 41598_2018_21694_MOESM5_ESM.pdf]

Explanations for Movies

(1) Movie 1--roi=1400-N=2-T=100K during [10, 14]ns
(roi=ρ, density of argon with unit of kg/m^3)

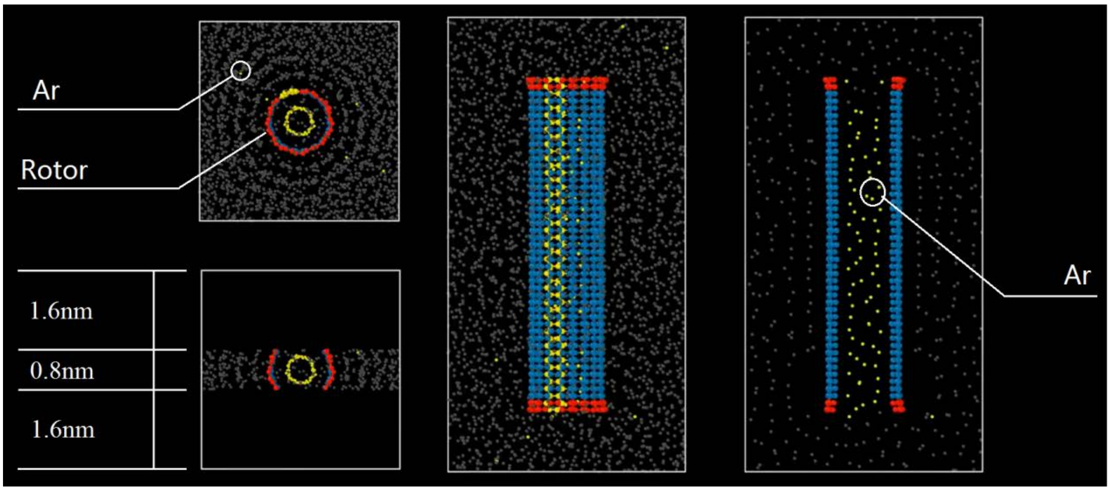

Ex-Figure 1. The rotation of rotor in argon with density of 1400 during [10, 14]ns. At 10ns, the argon atoms in the inner tube/rotor are labeled with yellow particles. To show the relative rotation of rotor, four columns of carbon atoms on rotor are labeled with yellow as well. When the argon atoms in yellow escape from the inner tube, or some argon atoms in grey enter into the inner tube, convection of argon exists. The lower left and the right subfigures show the atoms between two slices of Y=-0.4nm and Y=0.4nm. (With respect to Movie 1)

(2) Movie 2--roi=120-N=7-T=100K during [26, 26.1]ns at left, [31.9, 32]ns at right

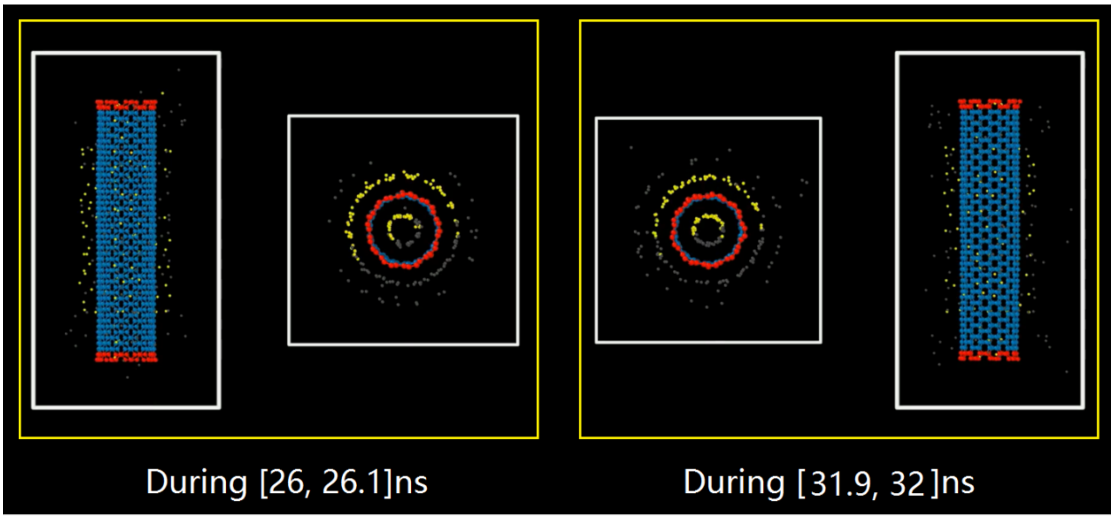

Ex-Figure 2. Comparison of rotational speed of rotor at different stages. Part argon atoms attached upon both inner and outer surfaces of rotor are labeled with yellow. (With respect to Movie 2)

(3) Movie 3--roi=400-N=2-T=100K during [10, 11]ns

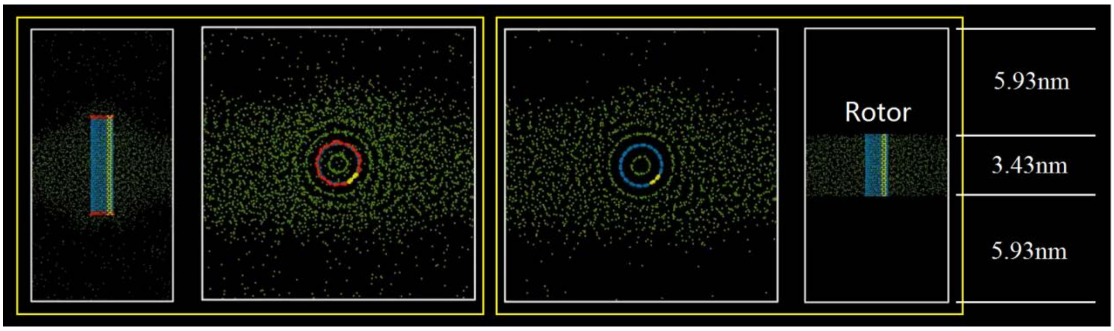

Ex-Figure 3. The cluster of argon nearby rotor at 100K. The left two subfigures are between two slices of Z=1.107nm and Z=4.55nm. (With respect to Movie 3)
